# Glucose-Sensing in the Reward System

**DOI:** 10.3389/fnins.2017.00716

**Published:** 2017-12-19

**Authors:** Laura L. Koekkoek, Joram D. Mul, Susanne E. la Fleur

**Affiliations:** ^1^Department of Endocrinology and Metabolism, Academic Medical Center, University of Amsterdam, Amsterdam, Netherlands; ^2^Laboratory of Endocrinology, Department of Clinical Chemistry, Academic Medical Center, University of Amsterdam, Amsterdam, Netherlands; ^3^Metabolism and Reward Group, Netherlands Institute for Neuroscience, Institute of the Royal Netherlands Academy of Arts and Sciences (KNAW), Amsterdam, Netherlands

**Keywords:** reward, nucleus accumbens, amygdala, SGLT, GLUT

## Abstract

Glucose-sensing neurons are neurons that alter their activity in response to changes in extracellular glucose. These neurons, which are an important mechanism the brain uses to monitor changes in glycaemia, are present in the hypothalamus, where they have been thoroughly investigated. Recently, glucose-sensing neurons have also been identified in brain nuclei which are part of the reward system. However, little is known about the molecular mechanisms by which they function, and their role in the reward system. We therefore aim to provide an overview of molecular mechanisms that have been studied in the hypothalamic glucose-sensing neurons, and investigate which of these transporters, enzymes and channels are present in the reward system. Furthermore, we speculate about the role of glucose-sensing neurons in the reward system.

## Introduction

Glucose is essential for the brain to function properly. It accounts for ~20–60% of total glucose consumption, depending on various factors such as physical activity, disease and energy status of the organism (Erbsloh et al., 1958). To ensure glucose availability, brain glucose levels are maintained around 2–2.5 mM (Silver and Erecinska, 1994). The brain continuously monitors circulating glucose levels and adequately responds to changes in glycaemia to keep glucose levels within this tight range. A critical brain mechanism that monitors glycaemia involves a specialized subset of neurons, which alter their activity in response to changes in extracellular glucose levels. These neurons, referred to as glucose-sensing neurons, were initially discovered in the ventromedial nucleus of the hypothalamus (VMH) (Oomura et al., 1969). The role of VMH glucose-sensing neurons, as well as glucose-sensing neurons in other hypothalamic nuclei, in the regulation of glycemia has been extensively studied (for excellent review see Steinbusch et al., 2015). However, glucose-sensing neurons have recently been identified in brain areas outside the hypothalamus, including in several brain nuclei that are part of reward system. The exact role of these extra-hypothalamic glucose-sensing neurons remains to be fully elucidated. Here, we review the current literature on the identification of glucose-sensing neurons in the reward system and the potential cellular mechanisms involved.

## The reward system and glucose-sensing

The reward system consists of multiple nuclei that play an important role in appropriate goal-directed behaviors by integrating aspects of reward, cognition, procedural learning, and motor control (Haber, 2014). A key axis of this circuit are the dopaminergic projections from the ventral tegmental area (VTA) and substantia nigra to the striatum. The ventral striatum, or nucleus accumbens (NAc), contains two primary subregions, the core and the shell. These subregions differ in their afferent and efferent connections and have partially overlapping, as well as distinct, roles in the reward-related behavior (Heimer et al., 1991; Zahm and Heimer, 1993; Carelli, 2004). Interconnected functional circuits with other nuclei, including the medial prefrontal cortex, hippocampus, amygdala, thalamus, lateral habenula (LHb), and lateral hypothalamus (LH) process motivational, associative, emotional, and affective information, thereby influencing the reward response (Kreitzer and Malenka, 2008; Haber, 2014; Stuber and Wise, 2016).

A vast amount of evidence supports a key role of dopamine in the reward-related behavior. Both natural and non-natural rewarding stimuli can modulate behavior through dynamic changes in dopamine signaling within the NAc (Johnson and North, 1992; Baik, 2013). However, other neurotransmitters, such as opioids and endocannabinoids, have also been implicated in reward-related behavior. Both opioid receptors and endocannabinoid receptors are expressed in the NAc and amygdala (Mansour et al., 1987; Herkenham et al., 1991). These different neurotransmitter systems modulate different processes in the reward system. In the response to food reward for example, a distinction can be made between “wanting” and “liking” of palatable food (Robinson and Berridge, 2003). Dopamine receptor 1 and 2 antagonists are capable of altering motivation to work for food, without altering the taste reactivity (a measure of liking) to food (Treit and Berridge, 1990; Aberman et al., 1998). In contrast, endocannabinoid and opioid signaling appear to play a role in the liking of food (Higgs et al., 2003) While the reward system is involved in responding to a wide variety of rewarding stimuli, this rewarding value of palatable food is of particular interest when studying glucose-sensing neurons in the reward system, as glucose is an important component of palatable food items. Food intake and the regulation of body weight is largely controlled by the hypothalamus. It integrates a variety of neuronal, hormonal, and nutrient-related signals to regulate these processes, thus serving as a homeostatic driver in the regulation of food intake and body weight (Schwartz et al., 2000; Stuber and Wise, 2016). The reward system on the other hand, modulates the hedonic control of feeding behavior, and can potentially override the homeostatic control of body weight (Lowe and Butryn, 2007). The reward system has therefore received much attention as a potential target for the treatment of obesity, and glucose-sensing neurons in the reward system could be an interesting component to study in this perspective.

## Glucose-sensing neurons in the reward system

To date, glucose-sensing neurons have been identified in several nuclei of the reward system. These include the NAc (Papp et al., 2007), amygdala (Nakano et al., 1986), LH (Yamanaka et al., 2003), hippocampus (Izumi et al., 1994), thalamus (Labouèbe et al., 2016), and medial prefrontal cortex (Mobbs et al., 2001). Although all these nuclei are part of the reward system, the NAc, amygdala and LH appear particularly important for the hedonic control of feeding behavior. We have previously hypothesized that an amygdala-VTA-NAc circuit is activated by the consumption of palatable foods containing both sugar and fat, and that this circuit is involved in the control of food intake (Mul and la Fleur, 2016). The LH receives input from both hypothalamic areas, such as the arcuate nucleus and the paraventricular nucleus (Broberger et al., 1998; Wu et al., 2015), as well as from nuclei in the reward system, including the NAc and amygdala (Heimer et al., 1991; Reppucci and Petrovich, 2016). The LH is thus perfectly situated to communicate with nuclei involved in homeostatic as well as hedonic feeding. We will therefore focus on glucose-sensing neurons in the NAc, amygdala, and the LH in this review.

### Nucleus accumbens

Glucose-sensing neurons in the NAc were initially identified using an *in vitro* application of glucose and subsequent measurement of changes in membrane potential. Using this procedure, two distinct types of glucose-sensing neurons were identified in the core and the shell of the NAc. In the shell, 27% of neurons that responded to changes in glucose showed increased activity upon glucose presentation, and were therefore labeled glucose-excited neurons. In contrast, 73% of the glucose-sensitive neurons in the shell of the NAc, showed decreased activity in response to glucose administration, and where thus labeled glucose-inhibited neurons. Surprisingly, an opposing pattern was found in the core of the NAc: 82% of total glucose-sensitive neurons were glucose-excited, whereas only 18% of all glucose-sensitive neurons were glucose-inhibited (Papp et al., 2007). It has to be noted, however, that although both glucose-excited and glucose-inhibited neurons were identified in the NAc, the majority of neurons (~75%) appeared to be non-responsive to glucose. Using a functional magnetic resonance imaging approach in rats, an increased BOLD signal was observed upon intra-gastric infusion of glucose (Tsurugizawa et al., 2008). An increase in BOLD signal suggests an increase in neuronal activity, as BOLD contrast imaging monitors the ratio of oxyhemoglobin to deoxyhemoglobin, which is larger when oxygen-rich blood flows to brain areas that are active. Whereas glucose drinking incites a state of satiety, administration of 2-deoxyglucose, a non-metabolizable analog of glucose, induces glucoprivation (Smith and Epstein, 1969). In line with the latter effect, subcutaneous injection of 2-deoxyglucose decreased BOLD signal in the NAc, suggestive of a general decrease in activity (Dodd et al., 2010). Finally, an immunohistochemical analysis in rats demonstrated increased expression of the early response gene *c-Fos* upon subcutaneous injection of 2-deoxyglucose, suggesting that a number of neurons in the NAc is activated in response to glucoprivation (Dodd et al., 2010). Of note, currently no clear immunohistochemical markers of neuronal inactivation are available. It thus remains possible that the majority of NAc neurons is inactivated by glucoprivation.

### Amygdala

An initial study in macaque monkeys identified glucose-sensing neurons in the amygdala using single neuron activity measurement during electrophoretic application of glucose. Of the 108 tested neurons, only 13 (12%) responded to glucose, and all of these neurons were glucose-inhibited neurons (Nakano et al., 1986). However, an *in vitro* study in rats identified glucose-excited as well as glucose-inhibited neurons in the amygdala. In this study, out of 522 neurons measured, 32 (6%) neurons were glucose-excited, while 39 (7.5%) neurons were glucose-inhibited (Zhou et al., 2010). Similar to findings in the NAc, the amygdala showed an increased BOLD signal following intra-gastric infusion of glucose, as well as a negative BOLD signal upon subcutaneous injection of 2-deoxyglucose (Tsurugizawa et al., 2008; Dodd et al., 2010).

### Lateral hypothalamus

The LH contains several neuronal populations, including two primary populations expressing the peptide melanin-concentrating hormone (MCH) or orexin, as well as smaller populations expressing GABA, glutamate, galanin or neurotensin. To date, orexin-expressing neurons and MCH-expressing neurons have been shown to have glucose-sensing properties. A study in mice revealed that LH orexin neurons are glucose-inhibited (Yamanaka et al., 2003). Orexin neurons in the LH are part of the reward system, as they are activated by cues associated with reward, including food and drugs of abuse (Harris et al., 2005). Moreover, an antagonist for the orexin receptor prevented cocaine-seeking in response to contextual cues (Aston-Jones et al., 2010). Orexin neurons are connected to the reward system through projections to the VTA, and can thereby directly influence dopamine signaling (Aston-Jones et al., 2009). MCH-expressing neurons in the LH on the other hand, appear to be exclusively excited by glucose (Burdakov et al., 2005). Moreover, *in vivo* analysis of these neurons revealed that glucose-sensing in these neurons plays an important role in the control of peripheral glucose homeostasis (Kong et al., 2010). As the receptor for MCH is highly expressed in the NAc (Saito et al., 1999), it has been hypothesized that MCH signaling directly influences food reward via the NAc (DiLeone et al., 2003).

Taken together, there is a variety of data to support the presence of glucose-sensing neurons in different nuclei of the reward system. We do have to note, however, that the properties of these glucose-sensing neurons have been tested using different *in vitro* methods of applying glucose to the neurons, and concentrations of glucose are used that are not within a physiological range. Thus, there is need for understanding glucose-sensing properties under physiological circumstances.

## Neuronal glucose-sensing machinery

Glucose-sensing neurons are equipped with specialized molecular machinery, which allows them to accurately sense and respond to changes in extracellular glucose. These glucose-sensing mechanisms were studied extensively in VMH neurons. For example, channels that transport glucose into the cell, enzymes involved in the translation of glucose metabolism to energy status of the cell, and transmembrane channels sensitive to changes in energy status, have all been studied with regards to their role in neuronal glucose-sensing. However, little is known about their relationship to glucose-sensing in neurons in the reward system.

### Glucose transporters

Members of the SLC2a family of transporters, better known as the glucose transporters (GLUTs), are the primary transporters responsible for uptake of glucose in the body. To date, 14 members of the GLUT family have been identified, of which 7 are expressed in the brain. Of these 7 transporters, GLUT1 and GLUT3 are primarily responsible for glucose uptake in neurons and glial cells. GLUT1 is expressed on brain endothelial cells and thereby facilitates glucose transport across the blood brain barrier (Dick et al., 1984). GLUT1 is also expressed on astrocytes (Morgello et al., 1995). In contrast, GLUT3 is predominantly expressed on neurons and is responsible for facilitating the majority of glucose transport into neurons (Leino et al., 1997). While GLUT1 and GLUT3 provide glucose to the brain, this does not mean that they are necessarily implicated with glucose-sensing. Indeed, all neurons require glucose as an energy substrate, but only a limited number of neurons alters their activity in response to changes in glucose. Regarding the pharmacological properties of different members of the GLUT family, GLUT2 appears to be a more suitable candidate for brain glucose-sensing. Due to its low affinity for glucose, GLUT2 transports glucose at a rate proportional to the extracellular glucose, providing direct information about the availability of extracellular glucose (Orci et al., 1989). GLUT2 is essential for glucose-sensing in pancreatic β-cells, and a large body of evidence supports the idea that it functions similarly within the hypothalamus. Furthermore, GLUT2 is expressed in parts of the brain stem, in the paraventricular nucleus of the hypothalamus (PVN), the LH and the arcuate nucleus of the hypothalamus (ARC), but, surprisingly, not the VMH (Leloup et al., 1994). Specific blocking of GLUT2 mRNA by antisense oligonucleotides either specifically in the ARC, or following non-specific intracerebroventricular infusion, produced a blunted increase in insulin upon a glucose bolus and diminished the increase in food intake after 2-DG administration, respectively (Leloup et al., 1998; Wan et al., 1998). These observations indicate that impaired hypothalamic GLUT2 function impairs the responses to changes in glycaemia, thus pointing toward a role for hypothalamic GLUT2 in glucose-sensing. Additional studies using several transgenic mouse models support the role of GLUT2 in the control of glycemia and food intake (Bady et al., 2006; Stolarczyk et al., 2010). Although neuronal GLUT2 appears to play a role in glycemia control, several studies indicate that astrocytic GLUT2 may be more important in glucose-sensing than neuronal GLUT2 (Guillod-Maximin et al., 2004; Marty et al., 2005). In the NAc of rats, GLUT2 protein is expressed in structures lining the ventricle, as shown by immunohistochemical staining (Arluison et al., 2004). However, a recent study found similar expression of GLUT2 surrounding the ventricle using a transgenic mouse model in which GLUT2 was linked to a fluorescent protein, but the authors showed that these fibers originated in the paraventricular thalamus (Labouèbe et al., 2016). Future investigations will unravel whether GLUT2 is really expressed in the NAc. In rats, expression of GLUT2 protein has been reported in the amygdala (Leloup et al., 1994; Arluison et al., 2004). Lastly, both mRNA (Leloup et al., 1994; Li et al., 2003) and GLUT2 protein have been found in the LH (Leloup et al., 1994; Arluison et al., 2004).

GLUT4 has also been implicated with glucose-sensing. GLUT4 is responsible for insulin-mediated glucose uptake in skeletal muscle, heart and adipose tissue, but is expressed in the brain as well. Brain GLUT4 might not function similar to peripherally expressed GLUT4, as neuronal glucose uptake is primarily mediated by GLUT3, which is more abundantly expressed than GLUT4. A role for GLUT4 in glucose-sensing is possible however, and this notion is supported by the observation that GLUT4 is expressed in 57% of glucose-excited neurons and 63% of glucose-inhibited neurons in the VMH (Kang et al., 2004). Moreover, brain-selective knockout of GLUT4 in mice negatively affected glucose-sensing, evident from the impaired counterregulatory responses to hypoglycemia (Reno et al., 2017). Although GLUT4 mRNA has been found in the NAc, several studies indicated the absence of GLUT4 protein in this brain region (El Messari et al., 1998, 2002). Weak to moderate GLUT4 staining has been reported in the amygdala, in particular in the basolateral amygdala (El Messari et al., 1998, 2002), while more pronounced expression of GLUT4 was observed in the LH (Leloup et al., 1996; El Messari et al., 1998, 2002). Thus, it seems unlikely that GLUT4 is involved in glucose-sensing in the NAc, but it cannot be excluded from investigations in the amygdala or the LH. Lastly, several additional GLUTs have been identified in the brain but limited studies have investigated their function. GLUT6, formerly named GLUT9, is expressed in the brain and, like GLUT2, appears to have low affinity for glucose (Doege et al., 2000). To date, no studies have been published showing expression patterns of GLUT6 in the brain, nor any investigations studying its function in the brain. GLUT8 is heavily expressed around the median eminence in the hypothalamus, but is also found in other areas, including the basomedial amygdala (Ibberson et al., 2002). Interestingly, whole-body GLUT8 knockout mice show increased locomotor activity, as well as increased emotional reactivity to a new environment (Schmidt et al., 2008). It is thus tempting to speculate that GLUT8, likely in the amygdala, is involved in this altered emotional reactivity, although this cannot be firmly concluded from a whole-body GLUT8 knockout mouse model. It is also important to note that GLUT8 is not expressed on the cell surface, but is present on intracellular vesicles. Whether GLUT8 is capable of functioning as a glucose sensor thus remains questionable. The final glucose-sensing candidate in the SLC2a family is GLUT10. GLUT10 is expressed in the several organs, including the brain (McVie-Wylie et al., 2001). Intraperitoneal injection of fluoxetine (a serotonin reuptake inhibitor) or pergolide (a dopamine receptor agonist), increases GLUT10 expression in the brain (Nagai et al., 2014). Unfortunately, this study analyzed gene expression in whole-brain samples, making it difficult to interpret these results. However, because the NAc contains high levels of dopamine receptors and receives input from serotonergic neurons, it would be particularly interesting to investigate if GLUT10 expression is specifically increased in the NAc after administration of either compound (Boyson et al., 1986). In summary, a number of GLUTs are expressed in the brain, of which several have been implicated with glucose-sensing. Additional research will be necessary to unravel the specific role of the different members of the GLUT family in glucose-sensing in the reward system.

### Sodium glucose cotransporters

In addition to facilitative transport by the GLUTs, sodium glucose cotransporters (SGLTs) also provide a mechanism for glucose entry into a cell. SGLTs transport glucose into the cell along a sodium gradient. To date, six SGLTs (SGLT1-6) have been identified. All of these, except for SGLT5, are expressed in the brain. However, the distribution of the brain-expressed SGLTs differs strongly and, unfortunately, not all brain SGLTs have been studied extensively. SGLT1 is the most studied of all SGLTs, and its expression in the brain has been confirmed in several studies (Poppe et al., 1997; O'Malley et al., 2006; Yu et al., 2010). Approximately 80% of SGLT-mediated glucose uptake in the midbrain is facilitated by SGLT1 (Yu et al., 2010). In rat hypothalamic neurons, 45% of the tested glucose-excited neurons were activated by a SGLT1-specific ligand, and conversely, the activation by glucose was suppressed by an SGLT inhibitor (O'Malley et al., 2006). Furthermore, reducing SGLT1 mRNA in the VMH, using short hairpin RNAs, improved the counterregulatory response to hypoglycemia, through enhanced glucose production in the liver. These findings clearly indicate that SGLT1 in the VMH is involved in glucose-sensing and the regulation of glucose homeostasis (Fan et al., 2015). Immunohistochemical analysis of SGLT1 protein showed expression in several brain regions, including the amygdala, but not in the NAc or LH. However, this study did not find SGLT1 expression in the VMH, which clearly contradicts with other studies (Yu et al., 2013; Fan et al., 2015). Thus, more studies will be necessary to clarify where SGLT1 is expressed in the brain. In addition to SGLT1, SGLT3 is also expressed in rat hypothalamic neurons in culture (O'Malley et al., 2006). Human DNA contains 1 gene coding for SGLT3 (*SLC5a4*), whereas mice and rats possess 2 genes coding for two SGLT3 proteins: SGLT3a and SGLT3b, and both these protein variants are expressed in hypothalamic neurons (Pletcher et al., 2000). Functional profiling of both SGLT3 subtypes revealed that SGLT3a, like human SGLT3, does not transport glucose across the cell membrane. However, unlike human SGLT3, binding of glucose to SGLT3a under physiological pH conditions did not lead to depolarization of neurons (Diez-Sampedro et al., 2003; Barcelona et al., 2012). Thus, whereas human SGLT3 has been proposed to serve as a glucose sensor, rodent SGLT3a might not be suitable. In contrast, SGLT3b does transport glucose across the membrane and has a similar low affinity for glucose as human SGLT3, which is favorable for a glucose sensor (Aljure and Díez-Sampedro, 2010). Human SGLT3 has been identified in the brain (Wright et al., 2011), but no studies have detailed where in the brain it is expressed. Thus, whether SGLT3(b) is involved in glucose-sensing in the reward system remains to be investigated in detail. To date, no studies have reported on the remaining SGLTs regarding their function or expression in the brain. SGLT2 has been investigated intensively as a therapeutic target for diabetic complications, as it plays an important role in the reabsorption of glucose in the kidney. In humans, SGLT2 RNA is only expressed in the cerebellum (Wright et al., 2011). In rats, SGLT2 mRNA has been found in the hippocampus, although this is only supported by unpublished data (Yu et al., 2010). Very low levels of SGLT4 mRNA have been detected in human brain tissue, raising the question whether SGLT4 plays a significant role in the brain (Tazawa et al., 2005). SGLT6, on the other hand, is expressed throughout the human brain. Strongest SGLT6 mRNA expression was found in the substantia nigra, although the amygdala and the NAc also showed moderate mRNA expression. Unfortunately, in this study the hypothalamus was only subdivided into the anterior and posterior part, with both areas showing equal and moderate mRNA expression (Chen et al., 2010). Furthermore, these data do not indicate whether the LH contains SGLT6 mRNA. In mice, whole-brain SGLT6 was tested, and equal levels of mRNA were found for SGLT6 compared to SGLT1 (Shah et al., 2012). In summary, the exact role of SGLT2, SGLT4 and SGLT6 in the brain remains to be fully determined.

Finally, we would like to highlight the sweet taste receptor, which like GLUTs and SGLTs binds glucose extracellularly, as a potential mediator of glucose-sensing. The sweet taste receptor, composed of the two subunits taste type 1 receptor 2 (T1R2) and taste type 1 receptor 3 (T1R3), is expressed in several organs including the tongue, pancreas and brain (Ren et al., 2009). A recent study has shown that the sweet taste receptor modulates glucose-sensing in glucose-excited neurons in the ARC (Kohno et al., 2016). To date, expression of this receptor has been demonstrated in the hypothalamus, hippocampus and cortex of mice (Ren et al., 2009), but we cannot rule out that future studies will reveal expression, and possible involvement, in glucose-sensing in the reward system.

Although both GLUTs and SGLTs transport glucose into a cell and are important for sensing, how they cooperate *in vivo* has not been studied in detail to date. It has been suggested, however, that due to the varying affinities, different transporters are important at different levels of glucose. For example, due to its high affinity for glucose, SGLT1 could serve as a glucose sensor during mild to moderate hypoglycemia (Fan et al., 2015). Moreover, SGLTs are electrogenic because of the small inward sodium current they produce when transporting glucose into the cell. Thus, SGLTs can mediate changes in activity, without the need for metabolizing glucose, thereby providing an additional mechanism by which glucose is sensed (Gribble et al., 2003). Future research will unravel how these two systems integrate glucose-related information under physiological circumstances.

### Energy status sensors

In addition to transporters responsible for the entry of glucose into cells, enzymes involved in glucose metabolism and energy status dynamics of the cell, have been studied for their role in neuronal glucose-sensing as well. In pancreatic β-cells, the enzyme glucokinase is responsible for metabolizing glucose. Glucokinase has an advantage over other hexokinases, because it is not inhibited by glucose-6-phosphate, the end product of the reaction it catalyzes. This indicates that the reaction rate of glucokinase depends solely on the availability of glucose, and is thus proportional to the available amount of glucose. Many studies have investigated the role of glucokinase in glucose-sensing neurons (for excellent review see Ogunnowo-Bada et al., 2014). Briefly, *in vitro* studies have demonstrated that glucokinase is involved in the majority of glucose-excited and glucose-inhibited neurons in the VMH. *In vivo* studies have revealed that activation of glucokinase in the VMH blunts the counterregulatory response to hypoglycemia (Dunn-Meynell et al., 2002). In contrast, inhibition of glucokinase improves the counterregulatory response to hypoglycemia, both by augmenting glucagon release, as well as by promoting feeding (Levin et al., 2008). Both *in situ* analyses and transgenic mice models, using a GFP protein expressed under the glucokinase promotor, have demonstrated that glucokinase is highly expressed in the amygdala and moderately expressed in the LH (Lynch et al., 2000; Stanley et al., 2013). No reports of glucokinase expression in the NAc have been published. However, glucokinase is only one of the enzymes involved in glucose metabolism that has been implicated with glucose-sensing. AMP-activated protein kinase (AMPK) which becomes activated when the AMP/ATP ratio in the cell increases, thus monitoring the energy status of the cell, has also been linked to glucose-sensing. Glucose-excited neurons isolated from the VMH require AMPK for appropriate glucose-sensing (Murphy et al., 2009). Furthermore, hypothalamic glucose-excited neurons *in vitro* become less responsive when AMPK is inhibited (Beall et al., 2012). *In vivo* experiments showed that, opposite to the effects on glucokinase, activation of AMPK enhances the counterregulatory response to hypoglycemia, and conversely, inhibition of AMPK blunts this response (McCrimmon et al., 2006, 2008). However, AMPK is widely expressed in many neuron populations, suggesting that it is not optimally suited as a marker of glucose-sensing. Nevertheless, evidence linking AMPK to glucose-sensing in the hypothalamus should not be ignored, and additional studies should include AMPK as a possible regulator of glucose-sensing in the reward system.

### Neuronal channels and transporters

Another category of proteins that has been explored in relation to glucose-sensing are channels that are involved in changes in neural activity upon fluctuation in available glucose. Based on glucose-sensing mechanisms in the pancreas, ATP-sensitive K^+^ channels have been studied intensively in the hypothalamus. ATP-sensitive K^+^ channels close when intracellular ATP levels rise, resulting in depolarization of the cell. The ATP-sensitive K^+^ channel consists of two subunits, the K^+^ inward rectifying channel (Kir6.1 or 6.2) and the sulfonylurea receptor (SUR1 or SUR2), which form a functional heterodimer. Expression of these components varies between different tissues. In pancreatic β-cells, predominantly Kir6.2 and SUR1 form the channel, whereas Kir6.1 and Kir6.2 and SUR1 and SUR2 are all expressed in the brain (Sakura et al., 1995; Davis-Taber et al., 2000; Li et al., 2003; Thomzig et al., 2005). Kir6.2 is predominantly expressed in neurons, whereas Kir6.1 is found only in a small population of neurons, and is primarily present on astrocytes (Thomzig et al., 2001, 2005). Specifically Kir6.2 has shown to be important for glucose-sensing in the VMH, as VMH neurons from mice lacking Kir6.2 no longer respond to increases in glucose (Miki et al., 2001). Both Kir6.2 and Kir6.1 are expressed in the NAc, whereas SUR1 appears not to be expressed (Karschin et al., 1997; Thomzig et al., 2005). No studies have currently reported on SUR2 expression in the NAc. The amygdala contains Kir6.2 and SUR1, and only very low levels of Kir6.1. In the LH, Kir6.2 has been shown to be essential for glucose-sensing in MCH-expresisng neurons (Kong et al., 2010). Kir6.2 is indeed expressed in the LH, but Kir6.1 shows greatest expression in this area (Thomzig et al., 2005). SUR1 is expressed in the hypothalamus at low levels, although detailed expression levels in the LH have not been specifically studied (Karschin et al., 1997).

Because Kir channels are sensitive to rises in ATP, they could play a role in glucose-excited neurons. However, different mechanisms would have to be present in glucose-inhibited neurons. One possible mechanism through which glucose-inhibited neurons function, was identified in studies investigating the orexin neurons in the LH. These neurons form an interesting population of glucose-inhibited neurons that are sensitive to extracellular fluctuations of glucose but do not depend on intracellular metabolism of glucose (González et al., 2008). In orexin neurons, the inhibition by glucose appears to be mediated by tandem pore K^+^ channels, in particular by the Twik1-related acid-sensitive K^+^ channel (TASK3), although TASK1 could not be completely ruled out as a contributor (Burdakov et al., 2006). In the NAc, only TASK3 mRNA has been observed, whereas the amygdala contains both TASK3 and TASK1 mRNA (Talley et al., 2001). Surprisingly, TASK3, TASK1 or TASK1/3 double knockout mice show unaffected glucose-sensing in orexin neurons (Burdakov et al., 2005; Guyon et al., 2009). Although compensation mechanisms could affect the observations in these knockout models, it remains questionable whether TASK1 or TASK3 are important for glucose-sensing.

In a recent elegant study, Chrétien and colleagues found that transient receptor potential canonical type 3 (TRPC3) channels are implicated with hypothalamic glucose-sensing. For example, unlike wild-type mice, TRPC3 knockout mice did not demonstrate inhibition of food intake in response to ICV injection of glucose. Insulin secretion in response to ICV injection of glucose was also blunted in these knockout mice. Lastly, although the number of glucose-excited neurons in the mediobasal hypothalamus did not differ between TRPC3 knockout and wild-type mice, neurons from mice lacking TRPC3 were significantly less responsive (Chretien et al., 2017). TRPC3 is expressed in striatal cholinergic interneurons, which are involved in complex processing of signaling in the striatum (Xie and Zhou, 2014). TRPC3 protein is also found in the amygdala in neonatal rats (Mizuno et al., 1999), while in adult rats TRPC3 mRNA has been reported to be present in the amygdala (Li et al., 1999). In conclusion, glucose-sensing neurons express a wide variety of proteins that can be part of the glucose-sensing machinery. This also highlights the possibility that extra-hypothalamic glucose-sensing neurons operate by different molecular mechanisms compared to those observed in the hypothalamus.

## Conclusions and perspective

Glucose-sensing is mediated by several glucose transporters, intracellular enzymes involved in the metabolism of glucose, and channels involved in the subsequent changes in neuronal activity. Different types of glucose-sensing neurons may function through specific combinations of these components of the glucose-sensing machinery. Alternatively, various mechanisms of glucose-sensing could operate side-by-side in one neuron. Future research will have to unravel how glucose-sensing occurs on a cellular level in the reward system, as well as the potential role for such glucose-sensing neurons in energy homeostasis.

From a clinical perspective, a full understanding of the central glucose-sensing mechanisms can be a potentially effective therapeutic target for patients with type I or type II diabetes mellitus. For example, recurrent hypoglycemia is a major side effect of insulin therapy and can lead to blunted counterregulatory responses to hypoglycemia (Diedrich et al., 2002). Notably, one study showed that recurrent hypoglycemia reduces sensitivity of VMH glucose-sensing neurons, and the authors hypothesized that this could contribute to the blunted counterregulatory responses to hypoglycemia (Song and Routh, 2006). While the hypothalamus has received the most attention for its role in glycaemia control, there is now evidence that indicates that the NAc also plays a role in glycemia control. Indeed, deep brain stimulation of the NAc shell increases glycemia, and this appears to be mediated through interactions with the LH (Diepenbroek et al., 2013). If the NAc influences glycemia under physiological circumstances, and whether NAc glucose-sensing neurons are involved in this process, remains to be investigated. Changes in glycemia have profound effects on feeding behavior. For example, infusion of 2-DG into the lateral ventricles, which induces central glucoprivation, promoted feeding in satiated rats (Berthoud and Mogenson, 1977). In addition, central infusion of glucose decreases feeding in mice (Cha et al., 2008). As such, glucose-sensing can directly convey important information about the energy status of the organism and influence behavior accordingly. Other signals that reflect energy status, are known to influence several neurotransmitter signaling pathways in the reward system. Ghrelin, a gastric hormone that stimulates feeding, can influence both dopamine and opioid signaling (Abizaid et al., 2006; Skibicka et al., 2012). Leptin, a hormone which functions as a satiety signal, can affect dopamine and endocannabinoid transmission (Di Marzo et al., 2001; Fulton et al., 2006). Thus, it is tempting to speculate that glucose-sensing conveys information about the energy-status as well, thereby influencing neurotransmitter activity in the reward system and subsequent behavior.

Diet-induced obesity induces profound adaptations in the brain, resulting in aberrant translation of the organism's energy status to appropriate metabolic behavior. Interestingly, glucose-sensing is also affected by obesity. For example, glucose-sensing in POMC neurons in mice fed a high fat diet for 20 weeks was impaired (Parton et al., 2007). Similarly, glucose-excited neurons from obese Zucker rats show abnormal changes in membrane potential in response to changes in extracellular glucose (Rowe et al., 1996). This study also found that glucose-excited neurons from obese Zucker rats lack a K_atp_ channel, necessary for glucose-sensing. Moreover, 8 weeks of a high-lard diet impaired hypothalamic glucose-sensing in rats, and lowered GLUT2 expression in the hypothalamus (de Andrade et al., 2015). To date, no reports exist on whether glucose-sensing in the reward system is altered in obese animals. However, function of the reward system itself is also affected by obesity. In humans, lower striatal dopamine 2/3 receptor binding has been found in obese subjects compared to lean individuals (Wang et al., 2001; van de Giessen et al., 2014). A similar observation was made for rats on a high-energy diet (van de Giessen et al., 2013). In rats that received a cafeteria style diet for 15 weeks, extracellular dopamine in the NAc was lower compared to controls (Geiger et al., 2009). The opioid system is also affected during obesity. Human obese individuals showed lower mu-opioid receptor binding in striatum compared to lean controls (Karlsson et al., 2015). Likewise, mice fed a high-fat diet for 15 weeks, showed decreased mu-opioid receptor expression in the NAc (Vucetic et al., 2011). Similar to the response by the opioid system, a down-regulation of CB1 in the hippocampus, cortex and NAc was seen in rats fed a high-fat diet (Harrold et al., 2002). Moreover, prepro-orexin mRNA was lower in obese rats compared to wild type (Cai et al., 2000), and narcolepsy patients which have orexin-deficiency, had a higher BMI than clinically similar patients without orexin-deficiency (Nishino et al., 2001). Likewise, overexpression of orexin induces a leaner phenotype (Funato et al., 2009). Lastly, MCH mRNA levels are increased in obese mice, and infusion of MCH in the lateral ventricle of the brain causes obesity (Qu et al., 1996; Gomori et al., 2003). Future research will have to investigate whether glucose-sensing in the reward system is also affected by obesity, but it is tempting to speculate that observed alterations in neurotransmitter functioning in the reward system during obesity, are related to possible obesity-induced changes in glucose-sensing in the reward system.

In conclusion, brain glucose-sensing appears to be a heterogeneous process, mediated by several potential candidates, including receptors, channels and sensors. While a vast amount of studies has investigated glucose-sensing in the hypothalamus, very little is known about the role of glucose-sensing neurons in the reward system. Applying our understanding of the different cellular mediators of glucose-sensing obtained in the hypothalamus to the glucose-sensing neurons in the reward system will aid in the characterization of glucose-sensing mechanisms in neurons of the reward system. This insight may identify new targets for the development of therapeutic treatments for recurrent hypoglycemia and metabolic disorders associated with impaired brain glucose-sensing.

## Author contributions

LK wrote the initial draft and JM edited and LK finished the review. SlF oversaw revisions and preparation of the final manuscript.

### Conflict of interest statement

The authors declare that the research was conducted in the absence of any commercial or financial relationships that could be construed as a potential conflict of interest.
